# Genome-wide association studies on malaria in Sub-Saharan Africa: A scoping review

**DOI:** 10.1371/journal.pone.0309268

**Published:** 2025-05-16

**Authors:** Morine Akoth, John Odhiambo, Bernard Omolo

**Affiliations:** 1 Strathmore Institute of Mathematical Sciences, Strathmore University, Ole Sangale Road, Nairobi, Kenya; 2 Division of Mathematics & Computer Science, University of South Carolina-Upstate, Spartanburg, South Carolina, USA; 3 School of Public Health, Faculty of Health Sciences, University of the Witwatersrand, South Africa; Clinton Health Access Initiative, Global Malaria Program, UNITED STATES OF AMERICA

## Abstract

**Background**: Malaria remains one of the leading causes of death in Sub-Saharan Africa (SSA). The scoping review mapped evidence in research on existing studies on malaria genome-wide association studies (GWAS) in SSA.

**Methods**: A scoping review was conducted to map existing studies in genome-wide association on malaria in SSA, with a review period between 1st January 2000 and 31st December 2024. The searches were made with the last search done in January 2025. The extracted data were analyzed using *R* software and *SRplot*. Relevant studies were identified through electronic searching of Google Scholar, Pubmed, Scopus, and Web of Science databases. Two independent reviewers followed the inclusion-exclusion criteria to extract relevant studies. Data from the studies were collected and synthesized using Excel and Zotero software.

**Results**: We identified 89 studies for inclusion. Most of these studies (n = 42, 47%) used a case-control study design, while the rest used cross-sectional, cohort, longitudinal, family-based, and experimental study designs. These studies were conducted between 2000 and 2024, with a noticeable increase in publications from 2012. Most studies were carried out in Kenya (n = 23), Gambia (n = 18), Cameroon (n = 15), and Tanzania (n = 9), primarily exploring genetic variants associated with malaria susceptibility, resistance, and severity.

**Conclusion**: Many case-control studies in Kenya and Gambia reported genetic variants in malaria susceptibility, resistance, and severity. GWAS on malaria is scarce in SSA, and even fewer studies are model-based. Consequently, there is a pressing need for more genome-wide research on malaria in SSA.

**Keywords**: Genome-wide association studies, malaria, Sub-Saharan Africa, scoping review.

## Introduction

Genome-wide association studies (GWAS) have become essential for identifying genes linked to human diseases, drawing significant interest from researchers worldwide [1]. Studying malaria in Sub-Saharan Africa (SSA) is especially critical, as the disease continues to pose a significant health threat and hinder socio-economic progress [2–6]. The World Health Organization (WHO) report estimated 599 thousand deaths in the year 2020, and 234 million cases in the year 2021 in Africa, accounting for 95% of the global malaria cases [7, 8]. GWAS have become powerful tools for understanding the genetic basis of complex diseases, including susceptibility, severity, and resistance to malaria [9–12]. These studies have illuminated the genetic basis of malaria-related traits by examining the entire genome for links between genetic variants and disease traits. This knowledge has paved the way for developing targeted interventions and personalized treatments [13]. In the context of SSA, where genetic diversity is exceptionally high [14], and the malaria burden is felt [9], GWAS holds immense promise for advancing our understanding of malaria epidemiology, pathogenesis, and treatment outcomes. A recent study by Abdellaoui and colleagues [15] reported that GWAS has significant potential, enabling discoveries that impact various fields, including population genetics, complex trait genetics, epidemiology, social science, and medicine.

Most GWAS have been conducted in Europe and Asia [16, 17], and Europe has reported bias in large-scale genomic studies. The international consortia for collaboration of genetic studies have left out Africa due to limited data and resources [18]. This has led many African countries to be unrepresented in the research despite the genetic diversity among African populations [14, 19, 20]. Despite all these, the field of genomics has advanced considerably due to a few current initiatives. Some of these initiatives are the 54Gene, The African Centre of Excellence for Genomics of Infectious Diseases (ACEGID), Inqaba Biotec (Africa’s Genomics Company), and The Human Heredity and Health in Africa (H3Africa) Consortium [21]. Ziyaad and colleagues [22], for instance, established the H3Africa Archive for African human genomic data management. This effort sought to improve data sharing and accessibility, helping to overcome the challenges of accessing high-throughput genomic technologies.

The primary goal of this scoping review was to map existing malaria GWAS studies in SSA. Despite notable progress in this field, a comprehensive synthesis of study findings is still lacking. This review aimed to highlight existing gaps and provide a foundation for future research recommendations. Previous research has focused on global perspectives, exploring the association of genetic variants with specific phenotypes. This study also looked into GWAS that employed model-based approaches to genetic association while addressing the key issues in malaria GWAS. The scoping review was chosen instead of a systematic review to map existing studies and provide an overview of the current state of malaria GWAS in SSA.

## Methods

### Study design

Four databases, namely Google Scholar, PubMed, Scopus, and Web of Science, were systematically searched to identify eligible studies published in English between January 2000 and December 2024. The year of study, study designs, subject areas, and countries of study were mapped and reported. A search strategy was developed to identify relevant literature using the Arksey and O’Malley [23] framework. The search terms used were “Genome-wide association studies”, “GWAS”, “ Malaria resistance”, “Malaria”, “Genetic association testing”, and “Sub-Saharan Africa”. All searches included peer-reviewed journal articles.

The study design aimed to conduct a comprehensive search for studies on GWAS in malaria within the SSA region. The Arksey and O’Malley framework has five steps: identifying the research question, identifying relevant studies, selecting studies, charting data, collating, summarizing, and reporting the data. The Preferred Reporting Items for Systematic Reviews and Meta-Analyses extension for Scoping Reviews (PRISMA-ScR) Checklist is in the S1 checklist [24].

#### Identifying research question.

The main research question for the scoping review was: What are the existing genome-wide association studies on malaria in SSA conducted between 2000 and 2024? Table 1 shows the population, concept, and context guide (PCC) used to assess the eligibility of the research question and to guide the selection of studies derived from the Joanna Briggs Institute [25].

**Table 1:** PCC framework used to determine the eligibility of the research question and to guide the selection of studies on GWAS in malaria.

*Population*	General population (adults and children, malaria vector)
*Concept*	Genome-wide association studies in malaria
*Context*	Sub-Saharan Africa

#### Identifying relevant studies.

Identifying relevant articles required a comprehensive search between 2000 and 2024 in four databases: Google Scholar, Scopus, PubMed, and Web of Science. These are life science journals with biomedical literature. Search strategies used on the four databases are available in the S1 File.

The articles went through title and abstract screening followed by full-text review by two independent reviewers. The two reviewers independently looked at each study to determine eligibility based on predefined inclusion and exclusion criteria. Disagreements between the reviewers were resolved through discussion to reach a consensus.

#### Search strategy.

A methodology was devised to examine the existing GWAS on malaria in SSA. Furthermore, the research sought to identify and investigate model-based studies that addressed the concept of heterosis. We searched the literature on malaria GWAS using the mentioned databases. The last search was conducted on January 21, 2025, and included all articles published up to December 31, 2024. A combination of the following keywords was used: “Genome-wide association studies”, “Malaria resistance”, “ Malaria”, “Genetic association testing”, and “Sub-Saharan Africa”. To refine the selection process, a research librarian from Strathmore University provided expert guidance on database selection, search term refinement, and search string construction. All retrieved articles were exported to Excel and Zotero software for screening, and the study selection process adhered to PRISMA-ScR guidelines [24].

#### Inclusion and exclusion criteria.

The criteria for inclusion of articles in the scoping review, provided in the S2 File, were as follows: the study must be a GWAS conducted in SSA between January 2000 and December 2024 and focused on malaria, the study must be published in English, and the study must have the full text available, study type included are the manuscripts and theses. The exclusion criteria included studies related to malaria that did not fall under the scope of GWAS, studies conducted in the regions of Algeria, Egypt, Morocco, Tunisia, and Libya, studies investigating non-malarial phenotypes, and systematic reviews, meta-analyses, and other reviews.

#### Selecting studies.

A sample of articles was initially reviewed in full to identify and gauge the themes of the studies. Emerging themes were noted, and an Excel spreadsheet was developed to highlight key variables of interest systematically. Two reviewers conducted the data extraction process independently, subsequently comparing their findings and reaching a consensus on the key variables to include in the study.

The titles of selected articles from various repositories were filtered and identified, and duplicates were removed using Excel software. Microsoft Excel was the primary data extraction tool employed, while Zotero served as the reference management software for extracting and organizing citation details. Microsoft Excel was also used to manage and chart extracted data. The two reviewers independently evaluated the abstracts of qualified articles based on predetermined inclusion and exclusion criteria and resolved any discrepancies. The database searches, keywords used, and the number of selected articles were noted.

Since this scoping review aimed to map primary research publications, systematic reviews, and review articles pertinent to malaria GWAS were noted during the search process but not included in the synthesis. The data was reported in the S3 File.

#### Charting the data.

Two independent reviewers extracted information by manually studying full articles, guided by the PCC framework. During extraction, the data focused on the study population (adults and children, malaria vector), concepts (malaria GWAS), and context (SSA). Information was extracted using the Excel software, including author names, publication dates, article titles, study designs, study country, study areas, genetic modes of inheritance for the model-based studies, genetic variants associated with malaria for some studies, and other significant findings. The PRISMA-ScR guidelines were applied to enhance transparency and reporting of the review process.

#### Collating, summarizing and reporting results.

Emerging themes related to GWAS in malaria were summarized. A descriptive analysis of peer-reviewed papers that addressed the research question was performed. The search covered various aspects, including the year of publication, the number of publications in different countries in SSA, the different study designs used, the categorization of subject areas into distinct categories such as susceptibility, severity, and resistance to malaria, the methodologies used to evaluate genetic association, drug resistance, population diversity, and host-parasite interactions. Discrepancies in extraction were resolved through discussion. Most primary authors explicitly specified the study designs in their publications. However, when the study designs were not stated, the review team carefully discussed the methodologies and context provided in the articles to determine and assign an appropriate study design classification.

Tables and graphs were employed where appropriate to represent the findings visually. *R* software was used to summarize and categorize the data. *SRplot* platform, a data visualization and graphing tool ( [26]), was also utilized to create the figures in compliance with the required standards. The scoping review results were used to identify knowledge gaps on malaria GWAS in SSA. The data and materials linked to this study are now available through the Open Science Framework (OSF) repository at https://doi.org/10.17605/OSF.IO/DFK5G.

## Results

**Fig 1:**
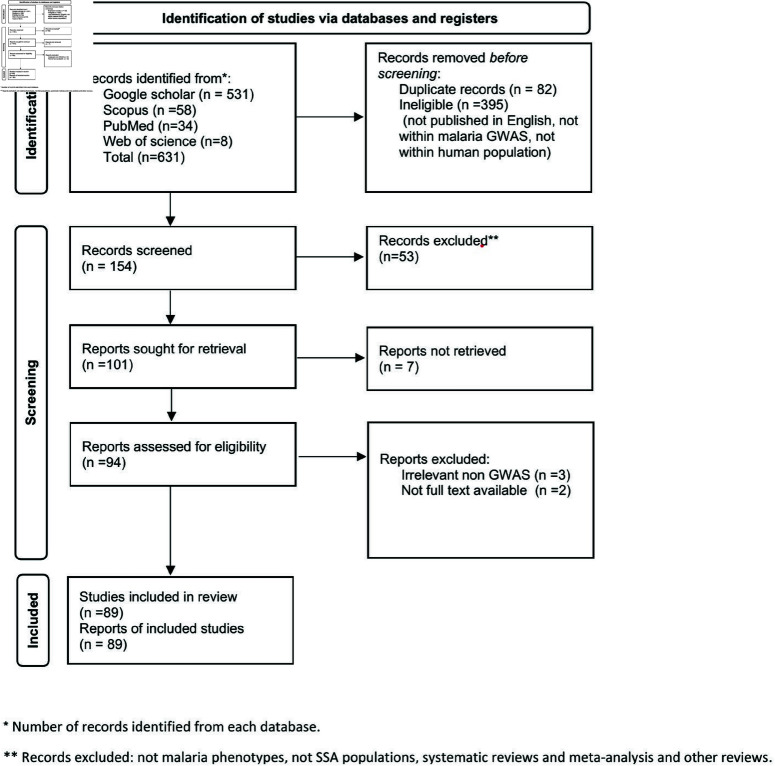
Preferred Reporting Items for Systematic Reviews and Meta-Analysis (PRISMA) on malaria GWAS in SSA.

A total of 631 studies were found in four electronic databases (Google Scholar (n=531), PubMed (n=34), Web of Science (n=8), and Scopus (n=58)). After removing 82 duplicate and 395 ineligible records for various reasons (not within the population, not malaria GWAS, and not published in English), 154 unique records underwent title and abstract screening. Of these, 53 records were irrelevant and excluded (16 non-GWAS, 12 non-malaria phenotypes, one not from the human population, 25 Systematic reviews and meta-analysis, and other reviews), leaving 101 reports for full-text retrieval. However, seven records were inaccessible or only had abstracts published, leaving 94 full-text reports for eligibility assessment by at least two reviewers. After full-text screening, five more records were excluded, and 89 studies were included in this scoping review. The study selection process is shown in Fig 1 [27].

### Study designs

**Fig 2:**
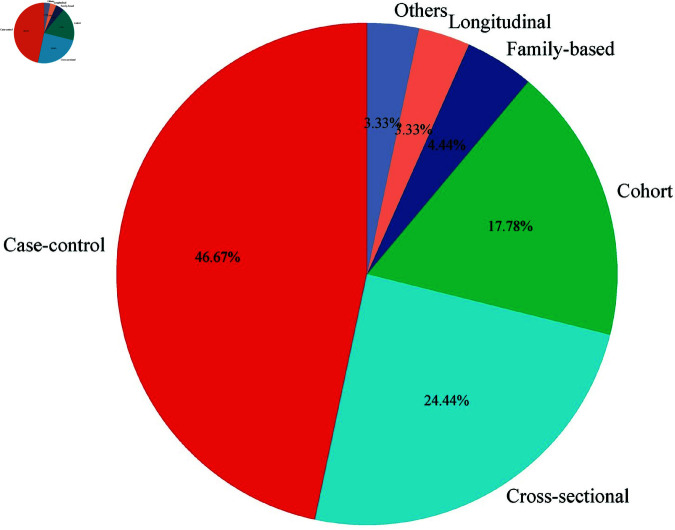
Different study designs used in malaria GWAS.

The predominant study design among the included investigations was case-control studies (n=42,47%) [8, 10, 28–45, 46–63]-67], cohort studies (n=16,18%) [68–83], cross-sectional (n = 23,24%) [60, 74, 79, 84–101]-103], longitudinal studies (n=3,3%) [96, 104, 105] and family-based studies (n=4,5%) [35, 106–108] (Fig 2). Other study designs include computational and laboratory-based experimental designs (n=3,3%) [93, 109, 110].

### Study areas

**Fig 3:**
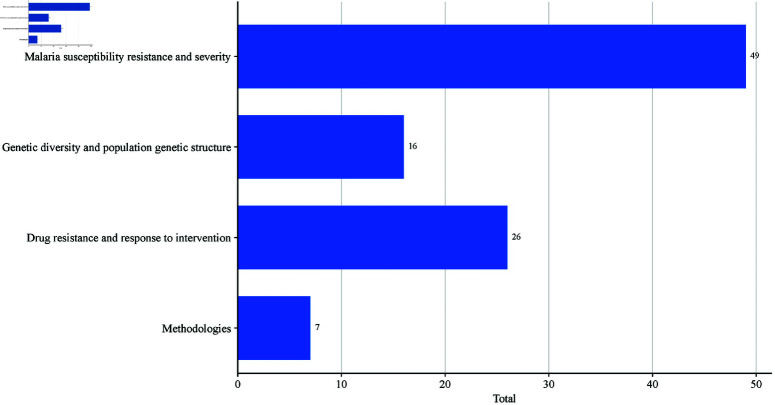
Different research areas in malaria GWAS in SSA.

Most GWAS on malaria focused on identifying genetic variants associated with susceptibility, resistance, and severity of malaria [8, 10, 28, 29, 31–39, 42, 43, 45–52, 54–59, 65–67, 69, 71–75, 77, 78, 81, 82, 84, 86, 92, 95, 96, 105, 108, 110]. Furthermore, considerable research had been conducted on methodological approaches to genetic association testing, malaria drug resistance patterns exhibited by malaria parasites, and the effectiveness of vaccine interventions [33, 41, 44, 47, 52–54, 63, 64, 68, 79, 80, 83, 87, 88, 90, 93, 94, 97, 101–104, 106, 107, 109]. Other areas of study included host-parasite interactions, population diversity and structures, genetic variation and evolutionary insights such as gene flow and natural selection [8, 17, 32, 60, 62, 67, 73, 74, 76, 86, 89, 96, 98–100, 108] and Mendelian randomization [91, 92] (Fig 3).

### Year of study and spatial distribution of studies

**Fig 4:**
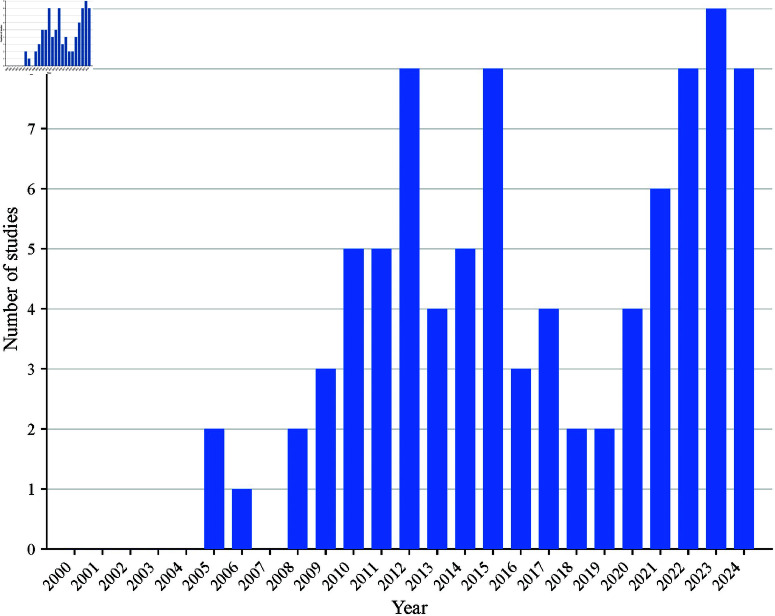
Number of articles in malaria GWAS in SSA published between 2000 and 2024.

Studies reported were published between 2000 and 2024, with a noticeable increase in publications from 2012, as shown in Fig 4.

**Fig 5:**
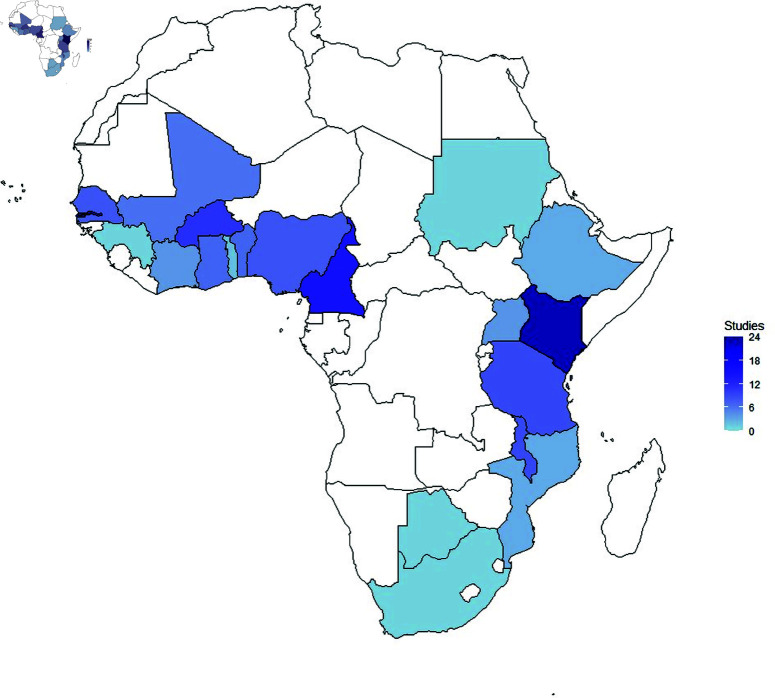
SSA countries conducting GWAS in malaria mapped with publications. The map was created using public domain data from Natural Earth (https://www.naturalearthdata.com/).

The study covered 21 different locations across various countries in SSA, with Kenya having the highest number of articles (n=23) [32, 36, 37, 41, 42, 47, 50, 51, 56, 62, 65, 73, 78, 81, 82, 90, 91, 93, 94, 104, 105, 109, 110] as shown in Fig 5. Other countries included Gambia (n=18) [28, 32, 35, 36, 39, 41, 43, 44, 46, 53, 54, 56, 65, 66, 73, 91, 108, 109], Cameroon (n=15) [30, 36, 41, 56, 64, 68, 70, 76, 77, 82, 85, 91, 98, 101, 102], Tanzania (n=9) [36, 38, 41, 56, 72, 75, 77, 90, 91], Ghana (n=7) [10, 52, 56, 60, 100, 103, 109], Malawi (n=9) [36, 41, 56, 62, 65, 73, 74, 87, 93], Burkina Faso (n=11) [36, 37, 41, 56, 69, 76, 82, 95, 97–99], Uganda (n=4) [47, 60, 62, 87], South Africa (n=1) [65], Togo (n=2) [89, 103], Mali (n=6) [41, 49, 57, 84, 95, 97], Benin (n=7) [48, 83, 87, 88, 92, 96, 103], Ethiopia (n=3) [77, 80, 86], Ivory Coast (n=4) [45, 76, 98, 103], Mozambique (n=3) [40, 55, 58], Nigeria (n=8) [36, 41, 54, 56, 76, 82, 98, 109], Guinea (n=1) [100], Botswana (n=1) [77], Senegal (n=8) [8, 59, 67, 76, 79, 93, 98, 107], Sudan (n=1) [108], and Democratic republic of Congo (n=3) [76, 90, 98]. There were no malaria GWAS in some SSA regions, such as Rwanda, Somalia, Chad, Namibia, Sierra Leone, Niger, Mauritania, Angola, Madagascar, and Burundi.

**Fig 6:**
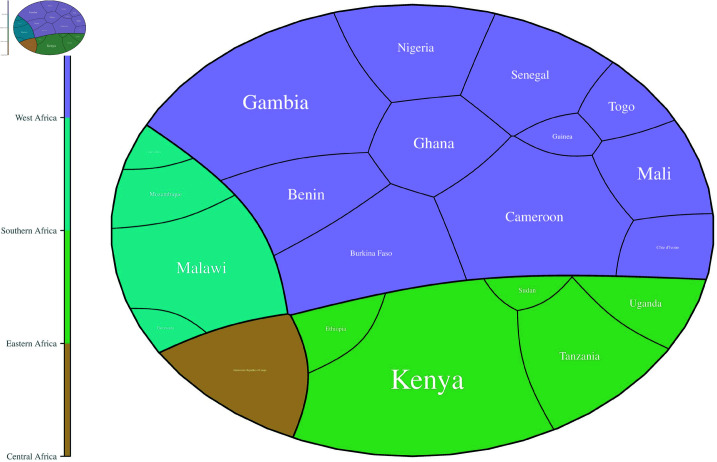
Polygon areas proportional to the number of studies conducted in SSA in different regions of Western Africa, Eastern Africa, Southern Africa, and Central Africa.

The research areas were further categorized into four distinct geographical regions, namely, Western Africa, Southern Africa, Eastern Africa, and Central Africa, as shown in Fig 6. Many articles were mapped in the Western Africa region, with Gambia having the highest number of articles (n=18).

### Genetic variants and malaria

Most studies focused on genetic variants associated with susceptibility, severity, and resistance to malaria (n=49). Table 2 shows some single nucleotide polymorphisms (SNPs) and genes associated with malaria protection and severity. Five studies [29, 38, 66, 84, 106] had genetic modes of inheritance on variants associated with malaria severity and protection. The studies are model-based, with heterosis as one of the genetic inheritance modes related to severe or mild malaria phenotypes. Ranvehall *et al*. [29] found SNP rs334 to be significantly associated with resistance to severe malaria. Manjurano *et al*. [38] reported the G6PD gene with heterozygous advantage effect, which has a deficiency known to protect against severe malaria in the Tanzanian populations. Maiga *et al*. [84] reported some of the regions of the genome under heterozygous advantage with significant SNPs associated with severe malaria. Other genetic models reported in the studies were the additive, recessive, and dominant modes.

**Table 2:** Associated SNPs and genes with protection and severe malaria with underlying genetic models in the studies

Author	Genetic model	Associated SNPs/ Genes	Malaria outcome
Jallow *et al*. 2009 [66]	Heterosis	rs10890361, rs316414, rs10249420	Severe
	Additive	rs5488069, rs2046784, rs2949632	Severe
	Dominant	rs10192428, rs12405994, rs16957052	Severe
	recessive	rs1384057 ,rs11013140, rs17728971	Severe
Maiga *et al*. 2014 [84]	Heterosis	rs4898389, rs7879049	Severe
	Additive	rs2515905, r1050828	Severe
	Dominant	rs915942	Severe
	Recessive	rs915941, rs1050828	
Manjurano *et al*. 2015 [38]	Heterosis	G6PD376, rs762515, rs2515905	Protection
Ravenhall *et al*. 2018 [29]	Heterosis	rs334, rs9296359, rs113449872	Severe
	Additive	rs149085856, rs17624383, rs2967790, rs144312177	Severe
	Recessive	rs3832816, rs8109875, rs6682413	Severe
Milet *et al*. 2016 [106]	Dominant	rs16942900 , rs3821869, rs12946796, rs10905522	Protection
	Additive	rs10757705, rs17640520, rs6675815, rs11679441, rs453079	Protection
	Recessive	rs971990, rs7694946, rs7123288	Protection

## Discussion

This scoping review aimed to identify existing malaria GWAS in SSA. A search was performed on Google Scholar, Pubmed, Scopus, and Web of Science, and the results are highlighted in Fig 1. It included 89 articles, with most studies covering susceptibility, resistance, and severity of malaria, where genetic variants associated with malaria have been discovered and provided vital information. The research showed increasing articles over time (Fig 4). In over 24 years, publications in malaria GWAS have consistently increased. The upward trend indicates that GWAS is increasingly recognized as a valuable tool for understanding the genetic factors that impact malaria susceptibility, resistance, and severity in SSA. A recent study by Abdellaoui *et al*. [15] reported an upward trend in GWAS publications worldwide between 2007 and 2022. Our findings reflect this trend in malaria GWAS in SSA, with a notable increase in research articles in 2012.

Most studies were conducted in Kenya and Gambia (Fig 5). The other countries with articles in malaria GWAS included Ghana, Cameroon, Tanzania, Burkina Faso, Benin, Uganda, Malawi, South Africa, Ethiopia, Ivory Coast, Senegal, Guinea, Congo, Mali, Sudan, Nigeria, Togo, Mozambique, and Botswana. Despite being malaria-prone regions, some countries had no studies reported on the subject area. These included Zambia, Rwanda, Somalia, Chad, Namibia, Sierra Leone, Niger, Eritrea, Liberia, Burundi, Central African Republic, and Lesotho. A review by Damena *et al*. [111] reported that malaria GWAS was conducted in specific regions due to genetic diversity across different countries. Regions with more significant heterogeneity in association signals were studied more frequently. The availability of resources also played an essential role in determining study locations within particular countries. Fig 6 further highlights the number of studies per region classified as Western, Eastern, Southern, and Central Africa. Many studies were reported in the Western region, with Gambia having the highest number of publications in the area. Previous studies have uncovered more than 3 million genetic variants from African populations, some considered novel [18]. The diverse genetic variants have been observed among different ethnic communities; hence, the potential to use knowledge to understand disease mechanisms and drug targets in clinical practices [17, 112]. Gouveia and colleagues [60] reported a high genetic diversity in Eastern Africa, while Ndo *et al*. [76] reported similar results in Western Africa. Although Africa is the continent with the highest genetic diversity, it has hosted only 2% of worldwide GWAS [18]. Studies on genetic diversity have shown that most GWAS focus mainly on European populations, as highlighted in studies such as Sirugo *et al*. (2019) [113] and Melzer *et al*. (2020) [114]. Despite Africa’s significant genetic diversity, GWAS have not adequately been conducted in SSA.

Most study designs were case-control, accounting for 47% of the articles (Fig 2). Case-control studies have been used to investigate disease risk factors and outcomes. They are less costly and less time-consuming than other study designs. The study design has been used in GWAS due to its efficiency and practicality in identifying genetic variants associated with complex diseases [115, 116]. In contrast to linkage studies, which require large sample sizes, case-control studies can detect genes that contribute only a minor fraction to the overall likelihood of a disease [117]. Heightened sensitivity is crucial in unraveling complex genetic associations. In addition, cohort, cross-sectional, longitudinal, family-based, and experimental study designs were reported.

Many studies have explored the genetic basis of malaria, focusing on susceptibility, severity, resistance to malaria, and associated genetic variants (Fig 3). Some GWAS have identified SNPs and genes linked to malaria outcomes under different genetic models (Table 2). Jallow *et al*. [66] reported the strongest association signals across dominant, trend, recessive, and heterozygous advantage models, reinforcing the complexity of genetic influences on malaria susceptibility. These findings align with broader GWAS evidence indicating that disease risk is modulated by multiple inheritance patterns [118]. The role of heterozygote advantage in malaria resistance has been documented in some studies [3, 118], and findings by Maiga *et al*. [84]. Manjurano *et al*. [38] further support the findings. Manjurano *et al*. [38] reported that female heterozygotes showed protective effects, suggesting the influence of sex-specific genetic mechanisms, which require further investigation. Similarly, other SNPs have been associated with malaria susceptibility under additive and recessive models, consistent with Ravenhall *et al*. [29] findings in the Tanzanian population. Some studies have consistently identified genetic variants following a heterotic mode of inheritance, linking them to malaria severity and protection [29, 38, 66, 84]. Similarly, Milet *et al*. [106] reported SNPs associated with malaria protection under additive, recessive, and dominant models in Senegalese children, reinforcing the genetic diversity underlying malaria resistance. These findings emphasize the complex genetic basis of malaria susceptibility, indicating that multiple genetic loci and inheritance patterns interact to affect disease outcomes [9, 73]. The consistency of these results across diverse African populations underscores the need for further replication studies to validate these associations and enhance disease risk prediction.

Additionally, investigating drug resistance patterns, refining methodological tools, and evaluating intervention effectiveness highlights the broad scope of GWAS. For example, studies by Milet *et al*. [106] looked at the GWAS of antibody response to malaria vaccines, and studies by Ali *et al*. [70] investigated the prevalence of drug resistance mutations among children in Cameroon. Several studies have used various statistical techniques, including Bayesian modeling [54] and generalized linear models [83], to explore genetic associations with malaria. Similar GWAS in other regions or those that address different phenotypes in SSA have adopted diverse methodologies [28, 119–124], to investigate genetic associations with the respective phenotype. In addition, a range of GWAS software tools, such as *IMPUTE2* as a framework used to impute genotypes, *bear* [109] and *R* [122], have been used to assess genetic correlations and explore associated genetic loci. A few studies on host-parasite interactions and evolutionary insights were also reported.

A key limitation of this scoping review is the relatively small number of GWAS studies focusing on malaria in SSA. This is mainly due to the region’s limited resources, infrastructure, and research funding, which reduced the number of studies available for inclusion. We extended our literature search to cover over 24 years to increase the number of articles. Secondly, the vast genetic diversity in SSA presents a significant challenge, as many studies fail to capture the genetic variations across different ethnic communities adequately. In addition, many countries within SSA reported fewer or no studies, which could lead to bias.

### Identified gaps in the review

The gaps identified in this study highlight the significantly fewer malaria GWAS than other studies globally. A study review by Abdellaoui and colleagues reported the existing GWAS between 2007 and 2022. In their findings, thousands of GWAS studies were reported, with a noticeable increase starting from 2011 [15]. However, malaria GWAS have remained limited in SSA, with the few existing studies conducted primarily in the continent’s western region. The global trend in GWAS has not been reflected in SSA.

Existing research has mainly focused on genetic variants associated with malaria susceptibility, severity, and resistance. Many of these studies have not examined the effects of different genetic modes of inheritance adopted in malaria research. This scoping review reported about 6% of model-based studies. Yet, certain genetic modes of inheritance, such as heterosis, in the previous studies have been linked with malaria resistance in patients with sickle cell anemia [118]. The impact of genetic modes of inheritance and the heterotic conditions on malaria and other phenotypes is yet to be explored.

Various genetic association tests, including the allelic test, MAX test, and Cochran-Armitage Trend Test (CATT), have been used to assess the relationship between genetic variants and malaria. In a previous study, the allelic test was reported to lose power when the genetic variant exhibited heterosis [125]; hence, the simulation study, under the SNP, showed that accounting for heterotic effects could enhance the power of the test, underscoring the need for model selection before genetic association testing.

## Conclusion

The findings in genetic studies on malaria in SSA highlight the significant efforts to deepen our understanding of malaria, including increasing publications, various methodological approaches, research on a diverse population, and the study of malaria GWAS in different geographic locations. However, despite the considerable genetic diversity observed in SSA, global representation in research still needs to be achieved. There is also a lot of untapped potential to develop novel methodologies and discover more genetic loci related to susceptibility, resistance, and severity of malaria.

The African Genome Variation project and the Africa BioGenome projects, among other projects, have enabled genetic studies in SSA by discovering millions of genetic variants [126, 127]. Such projects provide platforms for collaborations and partnerships among African scientists through biodiversity genomics.

## Supporting information

S1 Checklist PRISMA-ScR Checklist (DOC)

S1 File Search strategy. (DOC)

S2 File Inclusion and exclusion criteria. (XLSX)

S3 File Systematic reviews meta-analysis, and other reviews. (PDF)
