# Supplementary material for: Genome-wide association studies on malaria in Sub-Saharan Africa: A scoping review
Source: PLoS One. 2025 May 16;20(5):e0309268. doi: 10.1371/journal.pone.0309268 (PMC12083797; doi:10.1371/journal.pone.0309268)
Supplement: S1 File — (DOC) [file pone.0309268.s002.docx]

# **S1. Search strategy**

| **Database** | **Population** | **Context** | **Concept** | **Search Strings** | **Search Outputs** |
| --- | --- | --- | --- | --- | --- |
| PubMed | Human, Malaria vector | Sub-Saharan Africa | GWAS | ("Malaria”OR “Malaria resistance”) AND ("Sub-Saharan Africa") AND ("Genome-Wide Association Study" OR "Genetic Association Study") | 34 |
| Scopus | Human, Malaria vector | Sub-Saharan Africa | GWAS | ("Malaria”OR “Malaria resistance”) AND ("Sub-Saharan Africa") AND ("Genome-Wide Association Study" OR "Genetic Association Study") | 58 |
| Web of Science | Human, Malaria vector | Sub-Saharan Africa | GWAS | ("Malaria" OR “Malaria resistance”) AND ("Sub-Saharan Africa") AND ("Genome-Wide Association Study" OR "Genetic Association Study") | 8 |
| Google Scholar | Human, Malari vector | Sub-Saharan Africa | GWAS | ("Malaria" OR “Malaria resistance”) AND ("Sub-Saharan Africa") AND ("Genome-Wide Association Study" OR "Genetic Association Study") | 531 |
