# Supplementary material for: Genome-wide association studies on malaria in Sub-Saharan Africa: A scoping review
Source: PLoS One. 2025 May 16;20(5):e0309268. doi: 10.1371/journal.pone.0309268 (PMC12083797; doi:10.1371/journal.pone.0309268)
Supplement: S3 File — (PDF) [file pone.0309268.s004.pdf]

Systematic reviews and meta- analyses and other reviews in malaria GWAS in SSA

S3 File

There were 25 studies reported under systematic reviews (n=1), meta-analyses (n=8), and other reviews (n=17) in searches made between 2000 and 2024 as shown in Table 1.

Table 1: Systematic reviews, meta-analyses and other reviews

Studies	*Year*	*Study type*
MalariaGen [1]	2015	Meta-analysis
Gichohi et al. [2]	2016	Meta-analysis
Defo et al. [3]	2023	Meta-analysis
Donnelly et al. [4]	2016	Review
Band et al. [5]	2013	Meta-analysis
MalariaGen [6]	2019	Meta-analysis
Gibbs et al. [7]	2022	Review
Goheen et al. [8]	2017	Review
Driss et al. [9]	2011	Review
Damena et al. [10]	2021	Meta-analysis
Takem et al. [11]	2014	Review
Grant et al. [12]	2015	Review
Naing et al. [13]	2021	Meta-analysis
Aderoyeje et al. [14]	2024	Review
Kucharski et al. [15]	2024	Review
Adu-Agyarko et al. [16]	2024	Review
White et al. [17]	2024	Review
Rowell et al. [18]	2012	Systematic review
Arama et al. [19]	2018	Review
Kwiatkowski et al. [20]	2000	Review
Volkman et al. [21]	2017	Review
Venkataraman et al. [22]	2021	Meta-analysis
Volkman et al. [23]	2012	Review
Marquet et al. [24]	2018	Review
kwiatkowski et al. [25]	2005	Review

References

[1] Group MGENW. A novel locus of resistance to severe malaria in a region of ancient balancing selection. Nature. 2015;526(7572):253-7.

[2] Gichohi-Wainaina WN, Tanaka T, Towers GW, Verhoef H, Veenemans J, Talsma EF, et al. Associations between common variants in iron-related genes with haematological traits in populations of African ancestry. PLoS One. 2016;11(6):e0157996.

[3] Defo J, Awany D, Ramesar R. From SNP to pathway-based GWAS meta-analysis: do current meta-analysis approaches resolve power and replication in genetic association studies? Briefings in Bioinformatics. 2023;24(1):bbac600.

[4] Donnelly MJ, Isaacs AT, Weetman D. Identification, validation, and application of molecular diagnostics for insecticide resistance in malaria vectors. Trends in Parasitology. 2016;32(3):197-206.

[5] Band G, Le QS, Jostins L, Pirinen M, Kivinen K, Jallow M, et al. Imputation-based meta-analysis of severe malaria in three African populations. PLoS genetics. 2013;9(5):e1003509.

[6] Insights into malaria susceptibility using genome-wide data on 17,000 individuals from Africa, Asia and Oceania. Nature communications. 2019;10(1):5732.

[7] Gibbs KD, Schott BH, Ko DC. The awesome power of human genetics of infectious disease. Annual Review of Genetics. 2022;56(1):41-62.

[8] Goheen MM, Campino S, Cerami C. The role of the red blood cell in host defence against falciparum malaria: an expanding repertoire of evolutionary alterations. British journal of haematology. 2017;179(4):543-56.

[9] Driss A, Hibbert JM, Wilson NO, Iqbal SA, Adamkiewicz TV, Stiles JK. Genetic polymorphisms linked to susceptibility to malaria. Malaria journal. 2011;10:1-10.

[10] Damena D, Agamah FE, Kimathi PO, Kabongo NE, Girma H, Choga WT, et al. Insilico functional analysis of genome-wide dataset from 17,000 individuals identifies candidate malaria resistance genes enriched in malaria pathogenic pathways. Frontiers in Genetics. 2021;12:676960.

[11] Takem EN, Roca A, Cunnington A. The association between malaria and non-typhoid Salmonella bacteraemia in children in sub-Saharan Africa: a literature review. Malaria journal. 2014;13:1-13.

[12] Grant AV, Roussilhon C, Paul R, Sakuntabhai A. The genetic control of immunity to Plasmodium infection. BMC immunology. 2015;16:1-7.

[13] Naing C, Wong ST, Aung HH. Toll-like receptor 9 and 4 gene polymorphisms in susceptibility and severity of malaria: a meta-analysis of genetic association studies. Malaria Journal. 2021;20(1):302.

[14] Aderoyeje T, Erhuanga O. A Review on the Exploration of Genomic Approaches to Malaria Prevention and Treatment in Nigeria. Journal of Applied Sciences and Environmental Management. 2024;28(9):2937-41.

[15] Kucharski M, Nayak S, Gendrot M, Dondorp AM, Bozdech Z. Peeling the onion: how complex is the artemisinin resistance genetic trait of malaria parasites? Trends in Parasitology. 2024.

[16] Adu-Agyarko A, Etekochay MO, Gurajala S, Oduoye MO, Majumdar S. Malaria and neurological complications: intersecting mechanisms, disease models, and artificial intelligence-based diagnosis. Microbiology Independent Research journal. 2024;11(1):80-96.

[17] White N, Chotivanich K. Artemisinin-resistant malaria. Clinical Microbiology Reviews. 2024;37(4):e00109-24.

[18] Rowell JL, Dowling NF, Yu W, Yesupriya A, Zhang L, Gwinn M. Trends in population-based studies of human genetics in infectious diseases. PLoS One. 2012;7(2):e25431.

[19] Arama C, Quin JE, Kouriba B, Östlund Farrants AK, Troye-Blomberg M, Doumbo OK. Epigenetics and malaria susceptibility/protection: A missing piece of the puzzle. Frontiers in Immunology. 2018;9:1733.

[20] Kwiatkowski D. Genetic susceptibility to malaria getting complex. Current opinion in genetics & development. 2000;10(3):320-4.

[21] Volkman SK, Herman J, Lukens AK, Hartl DL. Genome-wide association studies of drug-resistance determinants. Trends in parasitology. 2017;33(3):214-30.

[22] Venkataraman GR, DeBoever C, Tanigawa Y, Aguirre M, Ioannidis AG, Mostafavi H, et al. Bayesian model comparison for rare-variant association studies. The American Journal of Human Genetics. 2021;108(12):2354-67.

[23] Volkman SK, Neafsey DE, Schaffner SF, Park DJ, Wirth DF. Harnessing genomics and genome biology to understand malaria biology. Nature Reviews Genetics. 2012;13(5):315-28.

[24] Marquet S. Overview of human genetic susceptibility to malaria: From parasitemia control to severe disease. Infection, Genetics and Evolution. 2018;66:399-409.

[25] Kwiatkowski DP. How malaria has affected the human genome and what human genetics can teach us about malaria. The American Journal of Human Genetics. 2005;77(2):171-92.
